# Ethanol induced oxidative stress, mitochondrial dysfunction, and autophagy in *Wickerhamomyces anomalus*

**DOI:** 10.1186/s12934-025-02864-0

**Published:** 2025-11-12

**Authors:** Xiaozhu Liu, Yujie Wang, Hongyue Xu, Lian Zhang, Yinfeng Li, Xuewen Zhang

**Affiliations:** 1 Guizhou Institute of Technology, Guiyang, 550000 China; 2 Hunan Agricultural University, Changsha, 410128 China

**Keywords:** Ethanol stress, Wickerhamomyces anomalus, Oxidative stress, Mitochondrial dysfunction, Autophagy

## Abstract

*Wickerhamomyces anomalus*, a flavor-modulating non-*Saccharomyces* yeast used in winemaking, experiences escalating ethanol stress during fermentation, yet the physiological and structural consequences remain unclear. This study assessed responses to 9% (v/v) ethanol stress with and without exogenous antioxidants (10 mM N-acetylcysteine and 2.5 mM glutathione) by measuring: reactive oxygen species (ROS) via fluorescence staining; superoxide dismutase (SOD) and catalase (CAT) activities plus glutathione content colorimetrically; mitochondrial membrane potential using rhodamine 123; ultrastructure via transmission electron microscopy; electron transport chain complex activities and ATP levels colorimetrically; and autophagy via monodansylcadaverine staining. Results indicated ethanol exposure induced ROS overproduction and oxidative stress while concurrently activating SOD and catalase activities and increasing GSH. Furthermore, ethanol reduced mitochondrial membrane potential, inhibited electron transport chain activity, decreased ATP synthesis, and triggered autophagy. Conversely, antioxidant supplementation alleviated oxidative damage, restored mitochondrial function, suppressed autophagy, and enhanced cell viability. These findings elucidate ethanol’s mechanistic toxicity in yeast and provide a foundation for developing antioxidant-enhanced, ethanol-tolerant *W. anomalus* strains for industrial fermentation.

**Supplementary Information:**

The online version contains supplementary material available at 10.1186/s12934-025-02864-0.

## Introduction

Yeast is widely used as the main “microbial cell factory” for producing both ethanol beverages and ethanol fuels due to its excellent ethanol productivity and tolerance [1]. However, yeast cells inevitably face increasing ethanol stress, particularly during the later stages of fermentation [2]. The accumulated ethanol induces excessive ROS production, causing oxidative damage to biomacromolecules such as proteins, lipids, and nucleic acids. This impairs cellular structural integrity, disrupts gene expression and metabolic processes, and ultimately leads to reduced cell vitality and increased autophagy [3]. Previous studies using *Saccharomyces cerevisiae* as a model organism have demonstrated that progressive ethanol accumulation during wine fermentation triggers oxidative stress in yeast [4, 5]. This stress damages plasma and mitochondrial membranes, promotes cell death, and ultimately reduces fermentation efficiency [6]. Therefore, alleviating ethanol-induced oxidative stress is essential for improving the survival of yeast cells and enhancing the fermentation efficiency in winemaking.

*Wickerhamomyces anomalus*, previously known as *Hansenula anomala*, *Pichia anomala*, and *Candida pelliculosa*, is a non-*Saccharomyces* yeast that can be isolated from diverse habitats and exhibits tolerance to a wide range of extreme environmental conditions, including pH 2.0-12.4 and temperatures from 3 °C to 37 °C [7]. *W. anomalus* has been shown to synthesize various glycosidases-including α-glucosidase, β-glucosidase, and α-mannosidase-which hydrolyze bound flavor precursors in fermentation substrates, promoting the release of volatile aroma compounds [8]. It is frequently used in mixed fermentations with *S. cerevisiae* to enhance wine flavor profiles [9]. For instance, when co-inoculated with *S. cerevisiae* in mixed fermentations or deployed for bioaugmentation during *Baijiu* solid-state fermentation, *W. anomalus* elevates ethyl acetate-a pivotal compound critically shaping *Baijiu* style and quality [10]. Similarly, in rice wine fermentation, this strain enhances aromatic complexity by boosting production of flavor-active compounds (esters, free fatty acids, alcohols) and amino acids, ultimately improving sensory evaluation scores [11].

In our previous study, the effects of ethanol stress on *W. anomalus* was examined and found that a high concentration of ethanol suppressed the growth of *W. anomalus* and damaged its cellular structure [7]. However, the physiological and structural alterations induced by ethanol in *W. anomalus* remain incompletely characterized, our study reveals that ethanol exposure triggers ROS accumulation and oxidative stress in this yeast. This response concomitantly enhances antioxidant enzyme activities and elevates glutathione (GSH) levels. Subsequent mitochondrial integrity impairment and functional disruption were observed, accompanied by induced autophagy. Notably, supplementation with antioxidants (N-acetyl-cysteine and GSH) mitigated ethanol-induced oxidative damage, restored mitochondrial function, suppressed autophagy, and ultimately improved cell survival. These findings delineate the mechanistic basis of ethanol toxicity in yeast and establish a foundation for developing antioxidant-enhanced, ethanol-tolerant *W. anomalus* strains for industrial fermentation.

## Materials and methods

### Yeast culture activation and treatment conditions

The yeast strain used in this study was *W. anomalus* C11, previously isolated from *Rosa roxburghii* Tratt fruit [12]. Cryopreserved cultures were revived by streaking onto YEPD agar (containing 1% yeast extract, 2% peptone, 2% glucose, and 2% agar) and incubating statically at 28 °C for 72 h.The activated cultures were inoculated into YEPD broth (1% yeast extract, 2% peptone, and 2% glucose) and incubated with shaking at 160 rpm for 8 h to obtain logarithmic-phase cells. Subsequently, a 5% inoculum from this culture was introduced into each treatment group. The experimental treatments were as follows: Ethanol Stress: Cells were exposed to 9% (v/v) ethanol. The control group was cultured under identical conditions without ethanol. Antioxidant Treatment: Cells were exposed to 9% ethanol and simultaneously supplemented with 10 mM N-acetyl-cysteine (NAC) and 2.5 mM glutathione (GSH). Antioxidative Enzyme Inhibitor Treatment: Cells were treated with either 100 µM 2-methoxyestradiol (2-ME; superoxide dismutase inhibitor) or 2 mM 3-amino-1,2,4-triazole (3-AT; catalase inhibitor) [13]. Rapamycin Treatment: Cells were treated with 0.5 µM rapamycin (Rapa). Mitochondrial Electron Transport Chain (mETC) Complex III Inhibitor Treatment: Cells were treated with either 0.2 µM myxothiazol alone or a combination of 9% ethanol and 0.2 µM myxothiazol. Following 6 h of incubation, cells from each group were harvested for subsequent experiments.

### Spot assay

Cell viability of *W. anomalus* was assessed by spot assay [7]. Following treatment, cells were harvested by centrifugation (5,000 rpm, 5 min), washed twice with distilled water, and resuspended to equal density (OD_600 nm_ = 1). Serial dilutions (10⁻¹ to 10⁻⁴) were prepared, and 2 µL aliquots spotted onto YEPD agar plates. After incubation at 28 °C for 36 h, colonies were documented using a digital microscope (Olympus, Japan).

### ROS determination

Intracellular ROS levels of *W. anomalus* cells were determined by measuring hydrogen peroxide and superoxide anion using fluorescent probes DCFH-DA (S0034S, Beyotime Biotech, China) and DHE (S0064S, Beyotime Biotech, China), respectively [14]. Qualitative analysis was performed using a fluorescence microscopy (Olympus BX51, Japan), while quantitative analysis was conducted with a fluorescence spectrophotometer (Hitachi F-4700, Japan). Both probes were used at working concentrations of 10 µM (DCFH-DA) and 4 µM (DHE) according to manufacturer protocols.

### Activities of superoxide dismutase and catalase determination

The activities of superoxide dismutase (SOD, EC 1.15.1.1) and catalase (CAT, EC 1.11.1.6) were determined using the Total SOD Assay Kit (A001-1, Jiancheng Bioengineering, China) and the CAT Assay Kit (A007-1-1, Jiancheng Bioengineering, China), respectively, according to the manufacturers’ protocols. Briefly, *W. anomalus* cells were collected by centrifugation at 6000 rpm for 10 min. The pellets were washed twice with phosphate-buffered saline (PBS, pH 7.4) and resuspended in 1 mL of PBS. The suspensions were sonicated using an ultrasonic cell disruptor at 150 W with a cycle of 5 s on and 5 s off for a total of 4 min, followed by centrifugation at 12,000 rpm for 10 min. The resulting supernatant was collected for enzyme activity assays. Then, 200 µL of substrate working solution was added, and the mixture was incubated at 37 °C for 20 min. Absorbance was measured at 450 nm for SOD activity and at 405 nm for CAT activity using a microplate reader. One unit of SOD activity was defined as the amount of enzyme that resulted in 50% inhibition, while one unit of CAT activity was defined as the amount decomposing 1 µmol of H_2_O_2_ per minute per milligram of protein.

### Concentrations of glutathione and malondialdehyde determination

The intracellular concentrations of reduced glutathione (GSH) and malondialdehyde (MDA) were measured using a commercial GSH and GSSG Assay Kit (S0053, Beyotime Biotech, China) and a MDA Assay Kit (A003-3, Jiancheng Bioengineering, China), respectively, according to the manufacturers’ instructions. Briefly, cells were centrifuged at 6000 rpm for 10 min, and the pellets were washed twice with PBS (pH 7.4) and resuspended in PBS. The suspensions were sonicated using an ultrasonic cell disruptor at 150 W (5 s on, 5 s off, for 4 min) and then centrifuged at 12,000 rpm for 10 min. The resulting supernatant was collected for subsequent assays.

For the GSH and GSSG assay, the supernatant was used directly for GSH measurement or treated with a GSH scavenger for GSSG determination. Then, 150 µL of total glutathione detection working solution and 50 µL of NADPH (0.5 mg/mL) were added, followed by incubation at 25 °C for 5 min. Absorbance was measured at 412 nm. Total GSH and GSSG levels were calculated using their corresponding standard curves. The GSH content was obtained by subtracting twice the GSSG content from the total GSH content, and the GSH/GSSG ratio was calculated by dividing the GSH content by the GSSG content.

For the MDA assay, the supernatant was processed according to the kit instructions, and absorbance was measured at 530 nm. The MDA concentration was determined using a standard curve.

### Mitochondrial membrane potential assay

Mitochondrial membrane potential (ΔΨm) was assessed qualitatively and quantitatively using the fluorescent probe rhodamine 123 (C2007, Beyotime Biotech, China) [15]. For qualitative analysis, fluorescence images were captured using a fluorescence microscope (Olympus BX51, Japan). Quantitative analysis was performed by measuring fluorescence intensity with a fluorescence spectrophotometer (Hitachi F-4700, Japan).

### Mitochondrial isolation and ultrastructural analysis

Mitochondria were isolated from ethanol-treated or antioxidant-supplemented cells using a commercial Cell Mitochondria Isolation Kit (C3601, Beyotime Biotech, China) according to the manufacturer’s protocol. Purified mitochondrial ultrastructure was examined by transmission electron microscopy (TEM). Briefly, cells were first collected by centrifugation (4,000 × g, 10 min), washed thrice with physiological saline, and then fixed in 2.5% glutaraldehyde. Subsequently, the fixed cells were washed three times with 0.1 M phosphate-buffered saline (PBS; pH 7.4). Following washing, the samples were dehydrated through a graded ethanol series (0%, 50%, 70%, 85%, 95%, 100%). The dehydrated samples were embedded in epoxy resin. Ultrathin Sects. (70–90 nm) were then cut using a diamond knife (LKB-Nova), stained with lead citrate, and ultimately examined under an FEI Tecnai Spirit TEM operating at 120 kV (FEI).

### Mitochondrial electron transport chain complex activity assay

The activities of mitochondrial electron transport chain (mtETC) complexes II (Succinate Dehydrogenase), III (Cytochrome bc₁ Complex/Coenzyme Q-Cytochrome c Reductase), and IV (Cytochrome c Oxidase) were assayed using the Succinate-CoQ Reductase Activity Assay Kit (BC3230, Solarbio, China), CoQ-Cytochrome c Reductase Activity Assay Kit (BC3240, Solarbio, China), and Cytochrome c Oxidase Activity Assay Kit (BC0945, Solarbio, China), respectively, following the manufacturer’s protocols.

Briefly, *W. anomalus* cells were collected by centrifugation at 6000 rpm for 10 min. The pellets were washed twice with PBS (pH 7.4) and resuspended in mitochondrial extraction buffer. The suspensions were sonicated using an ultrasonic cell disruptor at 150 W with cycles of 5 s on and 5 s off for a total of 4 min, followed by centrifugation at 12,000 rpm for 10 min. The resulting supernatant was collected for mitochondrial electron transport chain complex activity assays.

For the assay, the test sample was mixed with the corresponding detection reagent and incubated at 37 °C (for mtETC complex II) or 25 °C (for mtETC complexes III and IV) for 5 min. Absorbance was measured at 605 nm (for mtETC complex II) or 550 nm (for mtETC complexes III and IV). One unit of enzyme activity was defined as the amount of enzyme required to catalyze the production of 1 nmol of the corresponding product per minute per mg of protein. The enzymatic activities of mtETC complexes II, III, and IV were calculated accordingly for each group of cell samples.

### Activity of ATP synthase and concentration of adenosine triphosphate determination

Adenosine triphosphate (ATP) synthase activity was measured using the ATP Synthase Assay Kit (BC1445, Solarbio, China) according to the manufacturer’s instructions. The assay procedure was similar to that used for the mtETC complexes, with absorbance recorded at 660 nm. One unit of enzyme activity was defined as the amount of enzyme required to catalyze the production of 1 nmol of inorganic phosphorus per minute per milligram of protein.

ATP concentration was determined using the ATP Assay Kit (A095-1, Jiancheng Bioengineering Institute, China) following the manufacturer’s protocol. Cell samples were prepared using the same method as for the mtETC complex activity assay: cells were collected by centrifugation, disrupted by ultrasonication, and then centrifuged to obtain the supernatant. The supernatant was mixed with the detection reagent and incubated at 37 °C for 10 min. Absorbance was measured at 636 nm, and ATP content was calculated using the formula provided in the kit instructions.

### Autophagy determination

Autophagy levels in *W. anomalus* cells were assessed using the Autophagy Staining Assay Kit (C3018S, Beyotime Biotech, China) with the fluorescent dye monodansylcadaverine (MDC). Fluorescent images of cells from each treatment group were captured using an Olympus BX51 fluorescence microscope (Japan). Subsequently, cellular fluorescence intensity was quantified using a Hitachi F-4700 spectrofluorometer (Japan).

### Real-time quantitative PCR (RT-qPCR) analysis

Total RNA was extracted from *W. anomalus* cells using Trizol reagent (Invitrogen, USA), subsequently purified with a purification kit (Takara, Japan), and finally reverse-transcribed into cDNA using the PrimeScript RT Reagent Kit (Takara, Japan) as per the manufacturer’s instructions. RT-qPCR amplification was conducted on a LightCycler 96 system (Roche, Germany). Primer sequences for *ATG8* gene were forward 5‘-GTTCCTGCTGACCTTACCGT-3’ and reverse 5’-AGACATCAACGCCGCAGTAG-3’, *ATG11* gene were forward 5‘-ATCAAGCCTATGCCCTCGCCTT-3’ and reverse 5’-GCCAACCAAGGTTCGTCGTCAG-3’. Housekeeping gene *ACT1* was used as a reference gene, and its forward sequence were 5’-GGTACCACCATGTTCCCAGG-3’, and reverse 5’-ACGTTCTGGTGGAGCAATGA-3’.

The RT-qPCR protocol was performed using the following parameters: initial pre-denaturation at 95 ℃ for 30 s, followed by 40 cycles of denaturation at 95 ℃ for 10 s and annealing/extension at 60℃ for 30 s. A melting curve analysis was subsequently generated by heating the products from 95 ℃ for 10 s to 65 ℃ for 60 s, and finally to 97 ℃ for 1 s to verify amplification specificity. Gene expression data were normalized to the *ACT1* and analyzed using the 2^−ΔΔCT^ method.

### Statistical analysis

All of the tests were performed in triplicate. Data are presented as mean ± SD. Significance was determined by one-way ANOVA using SPSS 21.0(SPSS, Chicago, USA). *P* < 0.05 was considered significant.

## Results

### Ethanol induced overaccumulation of ROS and oxidative stress in *W. anomalus*

**Fig. 1:**
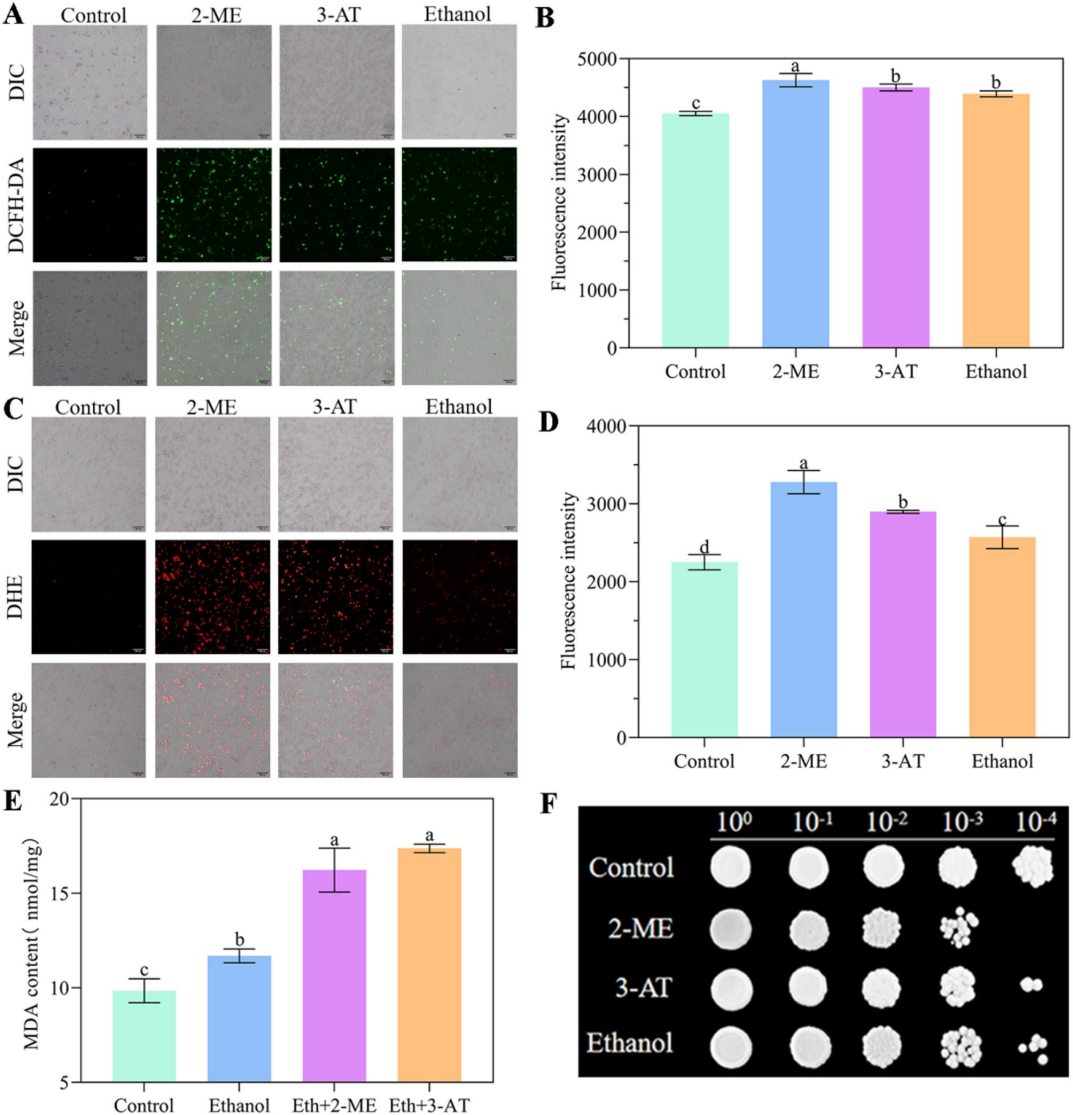
Ethanol-induced oxidative stress in *W. anomalus*. (A, B) Levels of H_2_O_2_ detected by DCFH-DA. (C, D) Levels of O_2_^·⁻^ detected by DHE. (E) Malondialdehyde (MDA) content. (F) Cell viability. Scale bar = 300 μm. Different lowercase letters above the error bars indicate significant differences (*P* < 0.05)

To assess ROS levels of *W. anomalus* under ethanol stress, cells treated with the antioxidative enzyme inhibitors 2-ME (SOD inhibitor) and 3-AT (CAT inhibitor) served as positive controls, while untreated cells served as the negative control. As expected, both inhibitors induced overproduction of H_2_O_2_ (detected by DCFH-DA; Fig. 1A and B) and O^2•⁻^ (detected by DHE; Fig. 1C and D), evidenced by significantly increased fluorescence intensities compared to the negative control group. Notably, ROS levels were also significantly elevated following ethanol treatment compared to the negative control (Fig. 1A and D). Furthermore, compared to the control group, MDA content increased significantly (Fig. 1E), and cell viability decreased markedly in the ethanol-treated group (Fig. 1F).

**Fig. 2:**
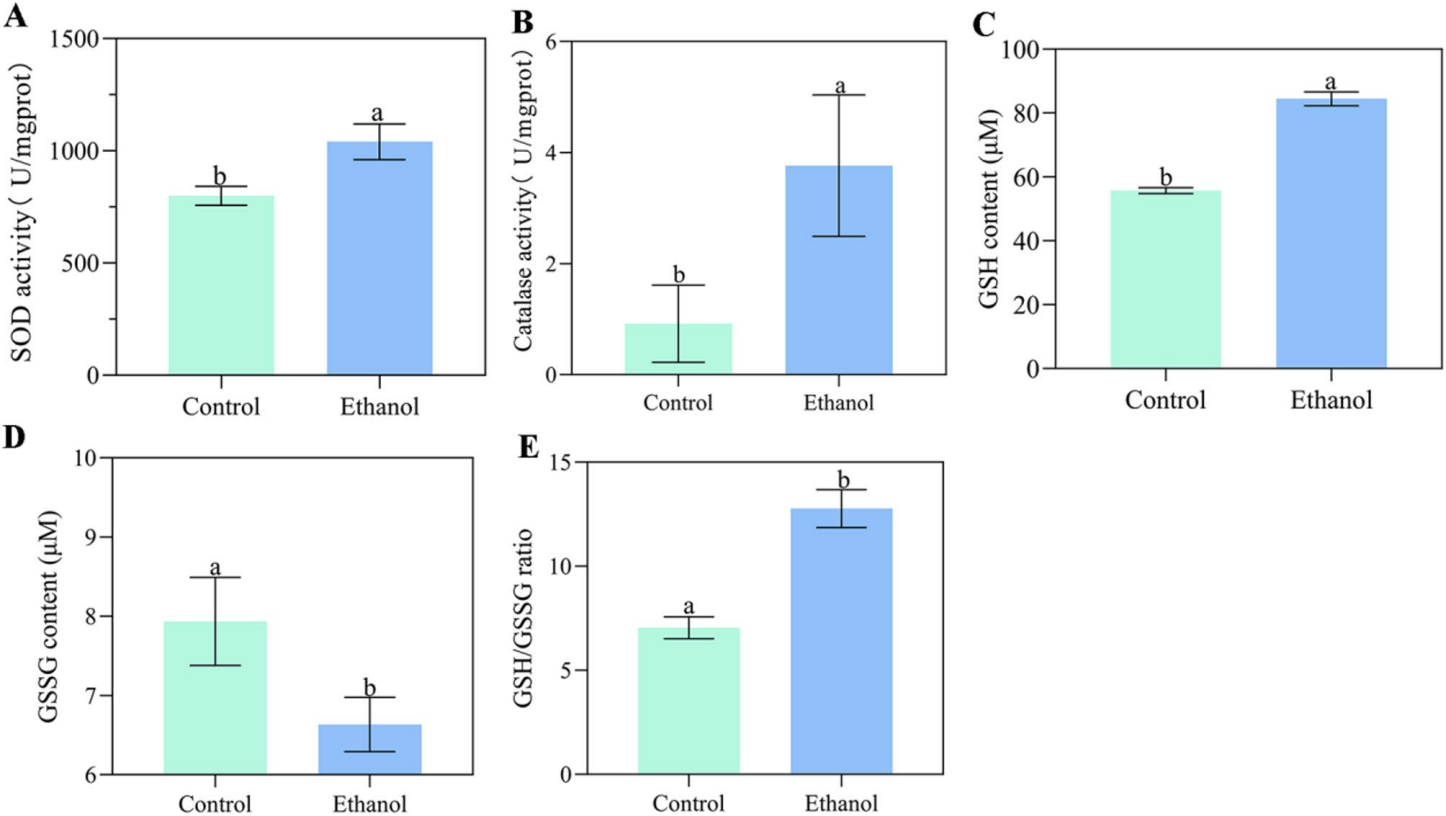
Activities of SOD (A) and CAT (B), contents of GSH (C) and GSSG (D), and the GSH/GSSG ratio (E) in *W. anomalus* under ethanol stress. Different lowercase letters above the error bars indicate significant differences (*P* < 0.05)

Activities of SOD and CAT were subsequently measured after ethanol treatment, revealing that ethanol stress significantly enhanced their activities (Fig. 2A and B). We also measured the contents of GSH and GSSG, and the GSH/GSSG ratio under ethanol stress. As demonstrated in Fig. 2C and E, the GSH content and GSH/GSSG ratio increased significantly, while the GSSG content decreased significantly.

Taken together, these data indicate that ethanol stress triggered excessive ROS production, leading to oxidative stress. This was accompanied by enhanced activities of antioxidant enzymes (SOD and CAT) and promoted synthesis of the antioxidant GSH, reflected in the increased GSH content and GSH/GSSG ratio.

### Ethanol induced mitochondrial dysfunction in *W. anomalus*

**Fig. 3:**
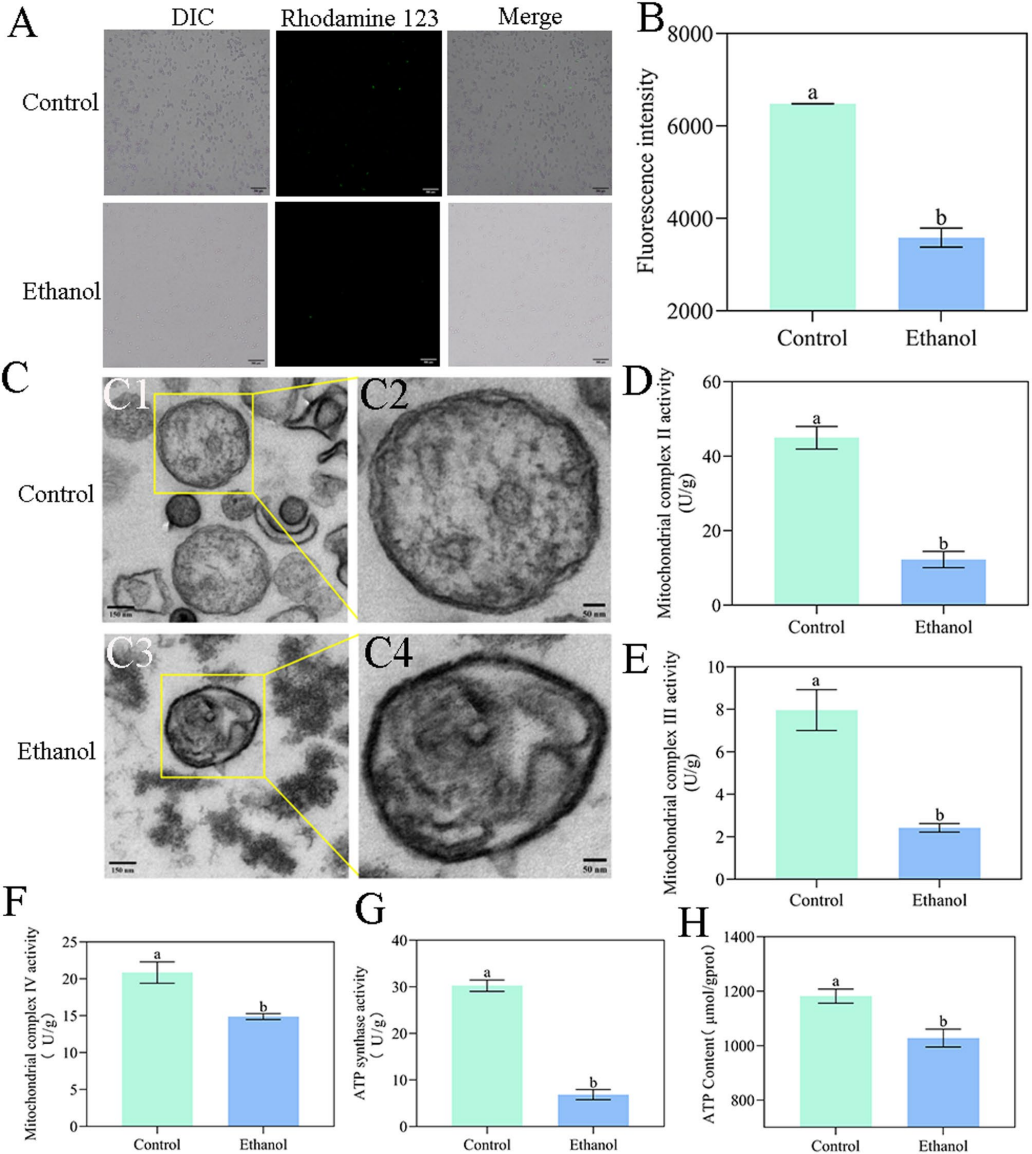
Ethanol-induced mitochondrial dysfunction in *W. anomalus*. (A, B) Mitochondrial membrane potential measured by rhodamine 123 assay. (C) Representative TEM images of *W. anomalu*s ultrastructure: Control cells (C1, C2) and ethanol-treated cells (C3, C4). Spherical mitochondria with intact double membranes and cristae (C1, C2) became elliptical with disrupted inner membranes and cristae (C3, C4) after ethanol stress. (D) mtETC complex II activity. (E) mtETC complex III activity. (F) mtETC complex IV activity. (G) ATP synthase activity. (H) Intracellular ATP content. Scale bars: A = 300 μm; C1, C3 = 150 nm; C2, C4 = 50 nm. Different lowercase letters above the error bars indicate significant differences (*P* < 0.05)

mtETC is reported as a primary site of ROS generation [16]. Given the observed ROS overproduction in *W. anomalus* under ethanol stress, we first investigated mitochondrial integrity by assessing ΔΨm (using rhodamine 123 assay) and ultrastructure (via TEM). Ethanol stress significantly depolarized mitochondria (Fig. 3A and B) and induced morphological alterations: spherical organelles with intact double membranes and cristae in control cells became elliptical and exhibited disrupted inner membranes and cristae following ethanol treatment (Fig. 3C). We further assessed mitochondrial function by measuring the activities of mtETC complexes, ATP synthase and intracellular ATP levels. Ethanol stress significantly reduced the activities of mtETC complexes II, III, and IV (Fig. 3D and F). Moreover, the activity of ATP synthase was also inhibited (Fig. 3G), resulting in decreased intracellular ATP content (Fig. 3H).

**Fig. 4:**
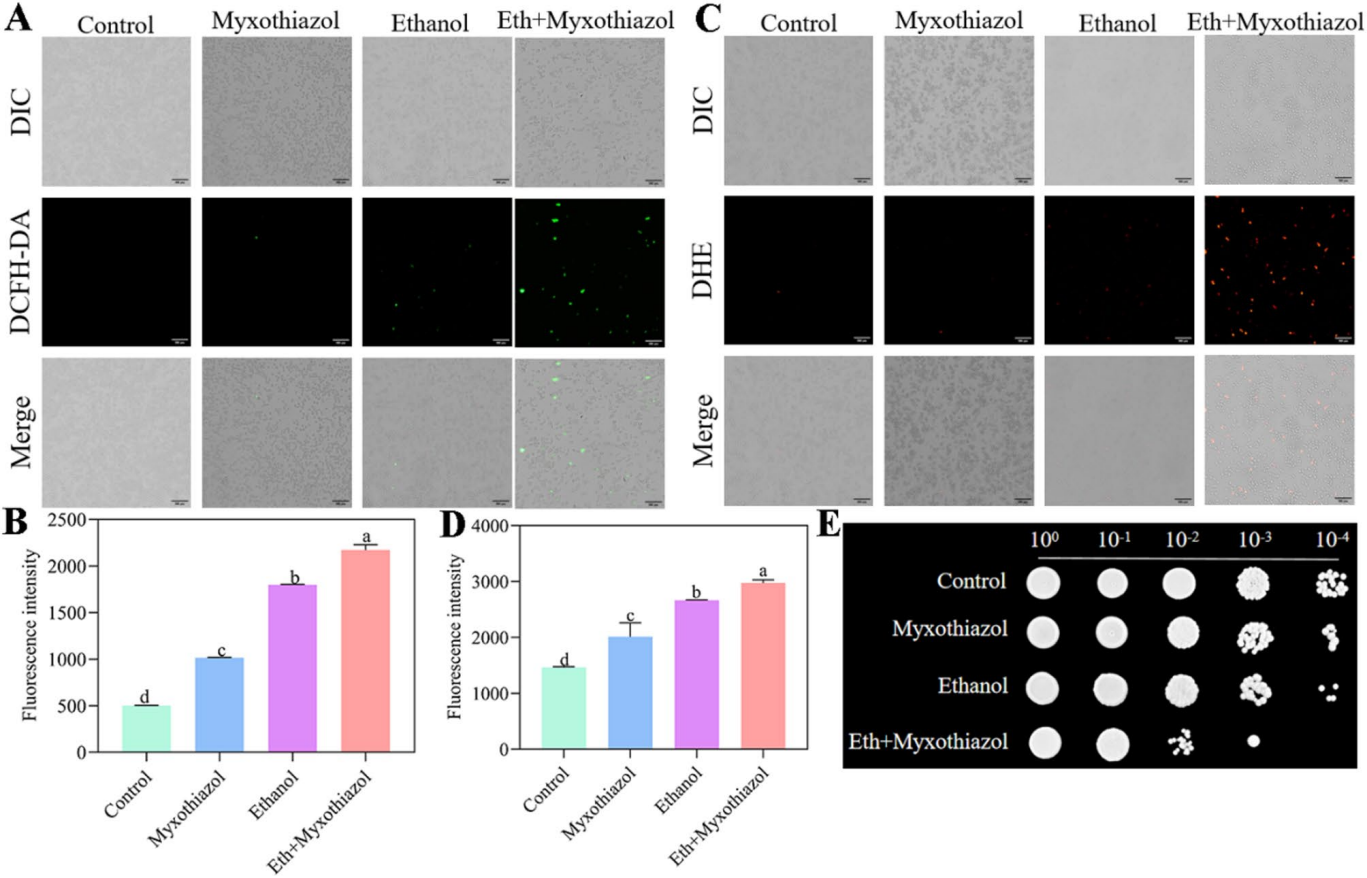
Myxothiazol increased production of H_2_O_2_ (A, B) and O_2_.^−^ (C, D)and reduced cell viability (E) of *W. anomalus.* Scale bar = 300 μm. Different lowercase letters above the error bars indicate significant differences (*P* < 0.05)

Additionally, myxothiazol-an inhibitor of mitochondrial complex III-was applied to cells with or without ethanol stress. The results demonstrated that, similar to ethanol treatment alone, myxothiazol significantly increased levels of H_2_O_2_ (Fig. 4A and B) and O_2_•⁻ (Fig. 4C and D). The combination of ethanol and myxothiazol synergistically elevated these ROS levels. Correspondingly, cell viability was markedly reduced in both ethanol- and myxothiazol-treated groups compared to controls, with the most severe decline observed under combined treatment (Fig. 4E). Collectively, these findings indicate mitochondria as the primary source of ethanol-induced ROS production in *W. anomalus*.

### Antioxidants alleviated ethanol-induced oxidative stress by restoring structure and function of mitochondrial

**Fig. 5:**
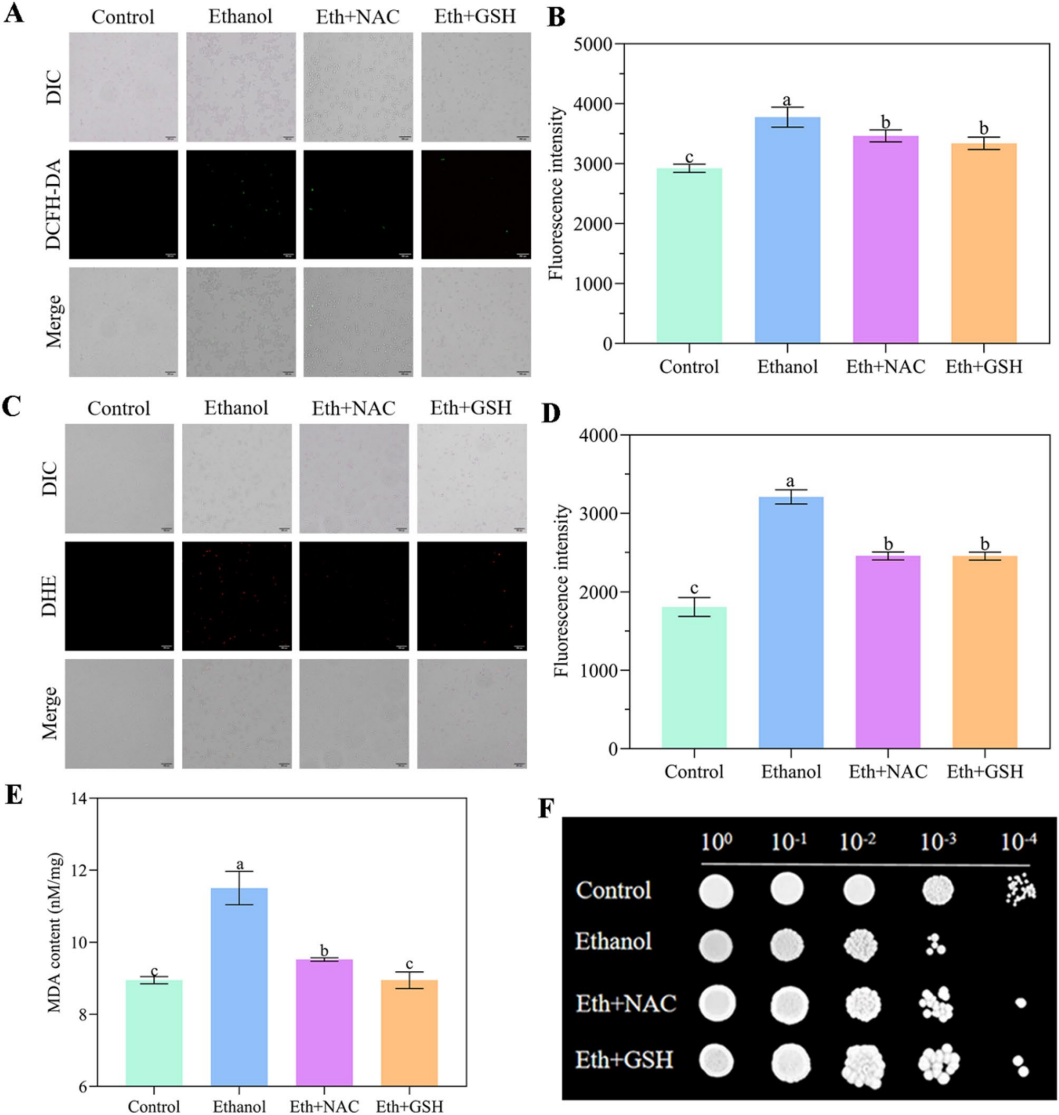
Antioxidants alleviated ethanol-induced oxidative stress in *W. anomalus*. (A, B) Levels of H_2_O_2_ detected by DCFH-DA. (C, D) Levels of O_2_^·⁻^ detected by DHE. (E) Malondialdehyde (MDA) content. (F) Cell viability. Scale bar = 300 μm. Different lowercase letters above the error bars indicate significant differences (*P* < 0.05)

To further confirm whether antioxidants could alleviated ethanol-induced oxidative stress, ROS production was measured in the presence of the antioxidants GSH and NAC under ethanol stress. As shown in Fig. 5A and D, excessive production of H_2_O_2_ and O_2_·⁻ was detected in the ethanol-treated group. In contrast, this excessive ROS production was partially reversed by the addition of either GSH or NAC under ethanol stress. Moreover, the content of the oxidative stress indicator MDA was significantly reduced in both the “Eth + GSH” and “Eth + NAC” groups compared to the ethanol-treated group (Fig. 5E). Cell viability was also significantly improved upon supplementation with GSH or NAC under ethanol stress(Fig. 5F). Interestingly, activities of SOD and CAT were significantly enhanced in both the “Eth + GSH” and “Eth + NAC” groups (Figure S1). These results demonstrate that antioxidants GSH and NAC mitigate ethanol-induced oxidative stress and promote cell survival of *W. anomalus.*

**Fig. 6:**
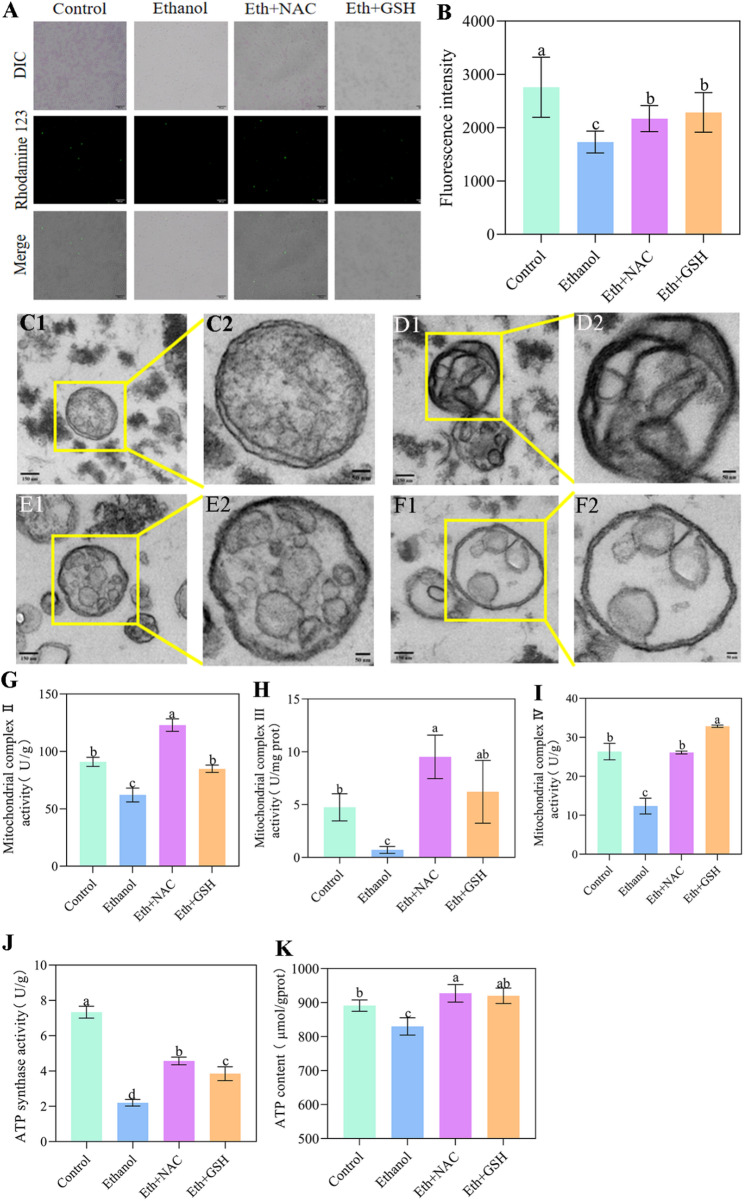
Antioxidants restore mitochondrial structure and function under ethanol stress in *W. anomalus*. (A, B) Mitochondrial membrane potential detected by rhodamine 123. (C1, C2) Control group ultrastructure; (D1, D2) Ethanol-treated group ultrastructure; (E1, E2) Ethanol + NAC-treated group ultrastructure; (F1,F2) Ethanol + GSH-treated group ultrastructure. (G) Mitochondrial complex II activity; (H) Mitochondrial complex III activity; (I) Mitochondrial complex IV activity; (J)ATP synthase activity; (K) Contents of ATP. Scale bars: c1, d1, e1, f1 = 150 nm; c2, d2, e2, f2 = 50 nm. Different lowercase letters above the error bars indicate significant differences (*P* < 0.05)

We further examined the mitochondrial structure and function of *W. anomalus* under ethanol stress in the presence of NAC or GSH. As shown in Fig. 6A and B, the reduced ΔΨm induced by ethanol was partially restored. Additionally, ultrastructural damage to mitochondria caused by ethanol treatment—such as disruption of double membranes and cristae—was ameliorated by NAC and GSH intervention (Fig. 6C1–6F2). Furthermore, the reduced activities of mETC complexes II, III, and IV were significantly increased when antioxidants were combined with ethanol treatment (Fig. 6G and I). Importantly, antioxidant treatment also improved ethanol-impaired mitochondrial function, as evidenced by enhanced ATP synthase activity (Fig. 6J) and increased ATP content (Fig. 6K).

### Ethanol stress induced autophagy in *W. anomalus*

**Fig. 7:**
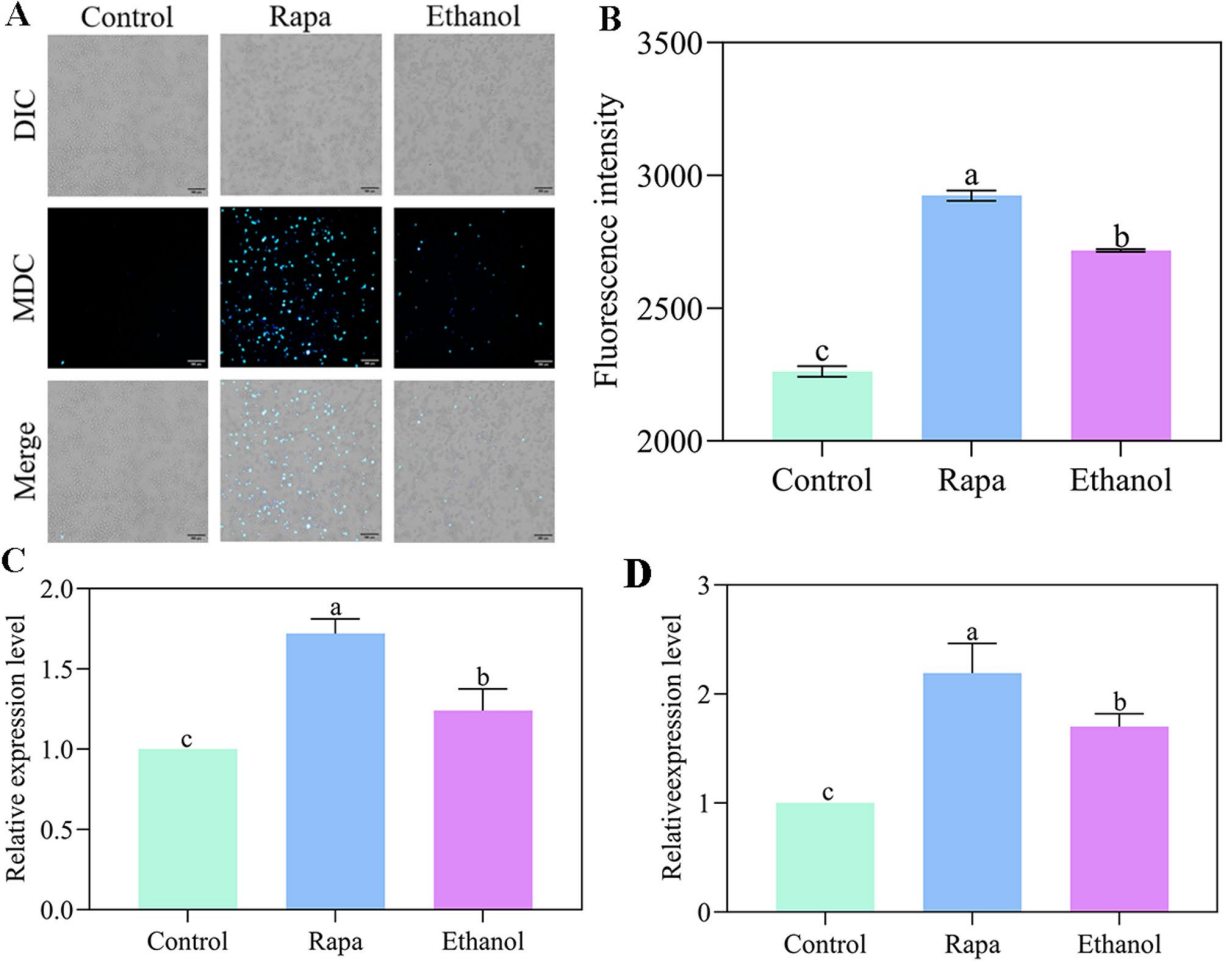
Ethanol stress induced autophagy in *W. anomalus*. (A, B) Autophagy levels detected by monodansylcadaverine (MDC) staining. (C) *ATG8* and (D) *ATG11* expression levels measured by RT-qPCR. Different lowercase letters above the error bars indicate significant differences (*P* < 0.05)

It was reported that elevated ethanol levels significantly impede cell growth, decrease viability, and trigger autophagy in *S. cerevisiae* [6]. To determine whether ethanol stress similarly induces autophagy in *W. anomalus*, we first measured cellular autophagy levels using the fluorometric assay, with the autophagy inducer rapamycin as a positive control. As shown in Fig. 7A and B, rapamycin treatment successfully induced robust autophagy formation, evidenced by a significant increase in fluorescence intensity. Notably, compared to the control group, ethanol-treated cells also exhibited significantly increased autophagy.

**Fig. 8:**
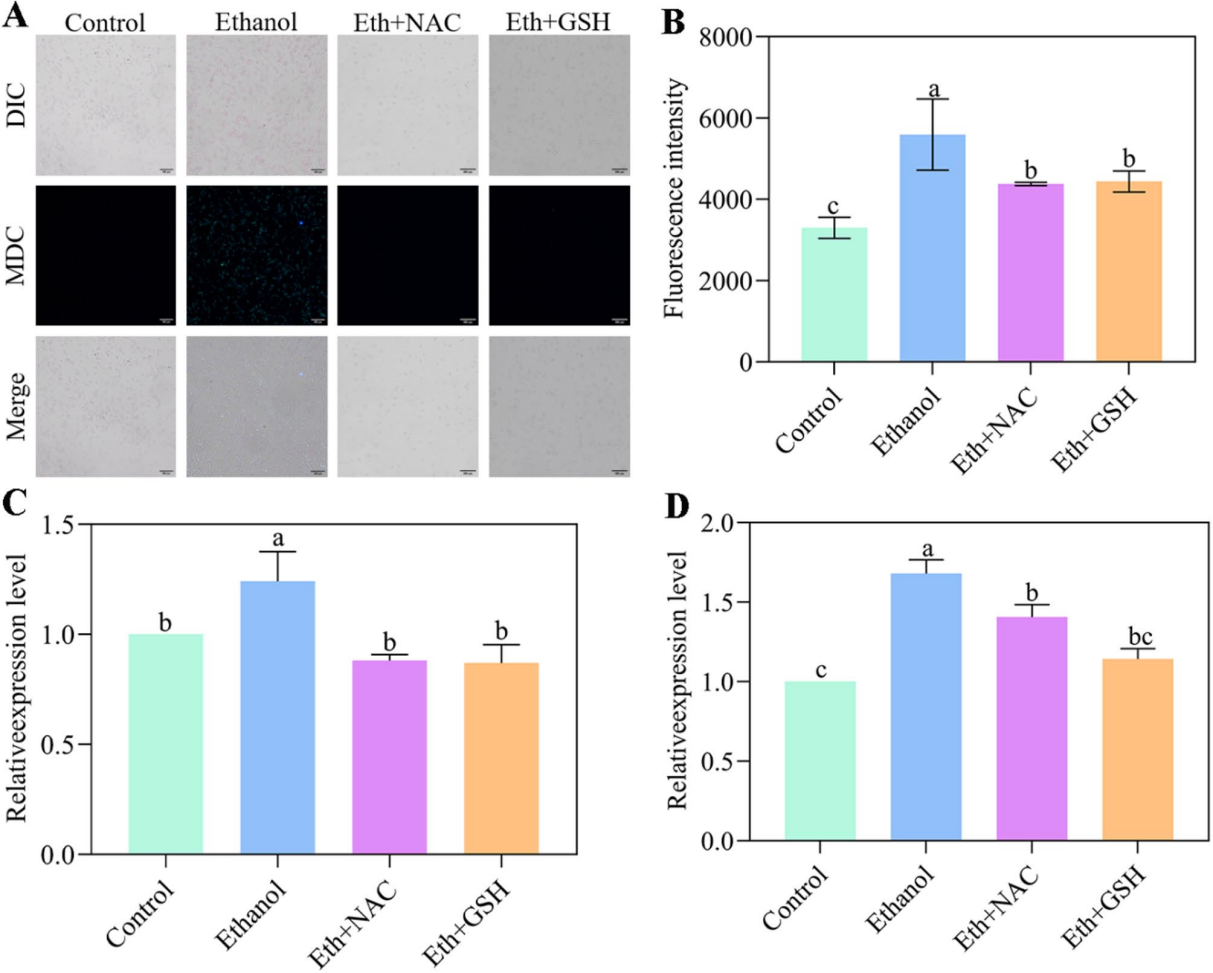
Antioxidants mitigate ethanol-induced autophagy in *W. anomalus*. (A, B) Autophagy levels detected by monodansylcadaverine (MDC) staining. (C) *ATG8* and (D) *ATG11* expression levels measured by RT-qPCR. Different lowercase letters above the error bars indicate significant differences (*P* < 0.05)

To further confirm ethanol-induced autophagy, we assessed the expression levels of key autophagy-related (ATG) genes using RT-qPCR [17, 18]. As expected, rapamycin treatment, a known autophagy inducer [19], markedly upregulated the expression of both *ATG8* and *ATG11* (Fig. 7c and d). Crucially, ethanol treatment also significantly increased the expression of *ATG8* and *ATG11* (Fig. 8C and D), indicating elevated levels of autophagy.

### Antioxidants mitigate ethanol-induced autophagy

The levels of cellular autophagy under ethanol stress, with or without simultaneous intervention by the antioxidants NAC and GSH, were first measured using a fluorometric assay. The results showed that ethanol significantly induced cellular autophagy in *W. anomalus*. However, following NAC and GSH intervention, autophagy levels were significantly reduced relative to the ethanol stress group (Fig. 8A and B). Furthermore, the expression levels of *ATG8* and *ATG11* were assessed. As shown in Fig. 8C and D, the addition of NAC and GSH significantly decreased the expression levels of both *ATG8* and *ATG11*, further indicating reduced levels of cellular autophagy.

## Discussion

Recently, *W. anomalus* has received increasing attention for its excellent flavor modulation capabilities and is frequently utilized in wine fermentation [20]. However, fermentation efficiency is closely associated with yeast cell vitality, which is negatively impacted by the gradual accumulation of ethanol during the process. It has been found that high ethanol concentrations at the end of fermentation can trigger ROS over-accumulation in *S. cerevisiae* cells, damaging cellular structures, disrupting metabolism, and potentially leading to incomplete fermentation [21]. In the present study, we found that exposing *W. anomalus* to 9% (v/v) ethanol induced ROS over-production (Fig. 1A and D, Fig. S1), which subsequently reduced physiological activity and cell vitality (Fig. 1F). Evidence indicates that MDA is one of the main products produced by yeast cells due to excessive eruption of ROS [22]. In this study, we also detected significantly elevated MDA levels in *W. anomalus* due to ROS overproduction under ethanol stress (Fig. 1E).

In yeast cells, excess ROS are removed to restore redox balance. ROS induce both SOD and CAT, which scavenge superoxide anions and convert H_2_O_2_ into H_2_O, respectively [23]. In this study, we found that ethanol-induced oxidative stress significantly activated intracellular antioxidant enzymes (SOD and CAT) (Fig. 2A and B)and increased GSH levels (Fig. 2C), which was consistent with ethanol treated *S. cerevisiae*. These suggests that enhancing antioxidant enzyme activity or antioxidant molecule concentrations through genetic modification or exogenous antioxidant supplementation may serve as an effective strategy to counteract ethanol-induced oxidative stress.

Mitochondria serve as the primary site for energy (ATP) synthesis in yeast cells through electron transfer and oxidative phosphorylation at the inner mitochondrial membrane [24]. However, environmental stressors can damage mtETC complexes, causing electron leakage that generates ROS [25]. Excessive ROS accumulation subsequently damages mitochondrial structure, inhibits mtETC complexes and ATP synthase activity, and induces mitochondrial dysfunction - ultimately amplifying ROS production [26]. In our study, ethanol-stressed *W. anomalus* exhibited decreased ΔΨm measured by fluorescent dye and showed inner membrane damage via TEM imaging (Fig. 3A and B). Furthermore, activities of mtETC complexes II, III, IV, and ATP synthase were significantly reduced in the ethanol-stressed group compared to controls(Fig. 3C and G), accompanied by decreased ATP levels (Fig. 3H). These results demonstrate that ethanol stress compromises mitochondrial integrity, leading to mitochondrial dysfunction in *W. anomalus*.

Oxidative energy conversion occurs via the four enzyme complexes comprising the mitochondrial respiratory chain, facilitating the oxidation of substrates-nicotinamide adenine dinucleotide (NADH) and flavin adenine dinucleotide (FADH_2_) [27]. Electron transfer from these substrates to molecular oxygen (O_2_), the terminal electron acceptor, drives respiratory chain activity [28]. This process couples redox reactions to proton extrusion at complexes I, III, and IV [29]. Evidence suggests that *S. cerevisiae* mitochondria lack a proton-translocating complex I, instead employing alternative dehydrogenases (e.g., Ndi1, Nde1, Nde2) [30]. Whether *W. anomalus* shares this characteristic remains unclear. Consequently, Complex I activity was not assayed in this study. Future work will assess dehydrogenase activities and expression levels of their encoding genes.

Glutathione, a tripeptide γ-glutamyl-L-cystinylglycine, is considered one of the most important cellular antioxidants, removing excessive ROS to maintain cellular redox stability [31]. It exists in two forms: reduced (GSH) and oxidized (GSSG) [32]. Under normal physiological conditions, glutathione is predominantly reduced due to the action of NADPH-dependent GSSG reductase. However, oxidative stress induced by environmental factors can decrease GSH levels, increase GSSG levels, and reduce the GSH/GSSG ratio [33]. Therefore, changes in GSH and GSSG levels, along with the GSH/GSSG ratio, serve as indicators of oxidative stress [34]. Our glutathione measurements revealed that ethanol-treated *W. anomalus* cells exhibited a significantly increased GSH content and GSH/GSSG ratio, along with an decrease in GSSG(Fig. 2C and E). This is consistent with the oxidative stress detected by fluorescence staining.

The decreased GSH levels induced by ethanol stress suggest that exogenous GSH supplementation may help alleviate oxidative stress in yeast cells. As expected, exogenous GSH addition efficiently alleviated ethanol-induced oxidative stress by reducing ROS production in *W. anomalus* (Fig. 5A and d, S2). Further investigation confirmed that GSH protected mitochondrial structural integrity, restored mitochondrial function, and enhanced cell viability(Fig. 6).

It should be noted that exogenous supplementation with the antioxidant NAC produced effects similar to those of GSH in mitigating ethanol-induced oxidative stress (Figs. 5 and 6). As a cysteine prodrug and widely used pharmacological antioxidant, NAC is reported to lower endogenous oxidant levels and protect cells against a wide range of pro-oxidative insults, including ethanol-induced stress [35, 36]. Although NAC itself is a weak direct scavenger of oxidants, its antioxidative mechanism is linked to the generation of hydrogen sulfide (H_2_S), a signaling molecule that has attracted considerable research interest due to its role in regulating cellular responses to stress [37, 38]. Furthermore, studies have found that *S. cerevisiae* synthesizes H_2_S during alcoholic fermentation, and strains with higher H_2_S production exhibit greater tolerance to oxidative stresss [39, 40]. This suggests that H_2_S may also protect yeast cells against ethanol stress.

Additionally, Autophagy is an evolutionarily conserved mechanism in eukaryotic cells that removes dysfunctional or unnecessary components and recycles intracellular nutrients [41, 42]. It can be activated under various stress conditions [43]. In this study, ethanol-stressed *W. anomalus* exhibited significantly increased autophagy levels, as measured using the fluorescent dye MDC-a response similar to that observed in *S. cerevisiae* (Fig. 7A and B). To date, 32 distinct ATG genes have been identified in yeast through genetic screening, all differentially expressed during autophagy [44]. Among these, we examined two key ATG genes, *ATG8* and *ATG11*, and found their expression significantly upregulated in ethanol-stressed *W. anomalus*(Fig. 7C and D). Notably, the expression of *ATG32*, which encodes the receptor protein for mitophagy (a selective form of autophagy), was also significantly increased(Fig. S2). These data demonstrate that ethanol stress induces autophagy in yeast cells, suggesting that this process may involve mitophagy.

Damage to the cell membrane, often indicated by excessive MDA production (a primary outcome of plasma membrane lipid peroxidation), can inhibit normal cellular functions including growth [45]. In our study, MDA levels in *W. anomalus* under ethanol stress were significantly higher than those in the control group, indicating compromised cell membrane integrity(Fig. 1E). This finding is consistent with our previous observations using transmission electron microscopy (TEM) (Fig. 3C) and will be further validated using the fluorescent probe propidium iodide to assess membrane integrity.

Inevitably, this study also has several limitations. First, although DCFA-DA and DHE are widely employed as classic ROS probes for detecting cellular oxidative levels across various systems, they suffer from non-specific reactions; for example, DCFA-DA can be influenced by elevated metal ion concentrations and enhanced peroxidase activity [46]. Although boronate-based and genetically encoded fluorescent probes offer greater specificity and are increasingly recognized by researchers, their commercial accessibility remains constrained [47]. Secondly, the drugs used in this study to induce ROS bursts—3-amino-1,2,4-triazole and 2-methoxyestradiol—are more commonly applied in mammalian cells, and while some studies have reported their use in yeast, further screening for compounds better suited to yeast models is necessary [48]– [13, 49]. Finally, MDA detection in this study relied on a qualitative thiobarbituric acid-reactive substances (TBARS) assay, whereas HPLC-based quantitative methods have been extensively reported and adopted [50], prompting the adoption of an integrated qualitative and quantitative approach in future research.

## Conclusion

Our results demonstrate that ethanol exposure induced ROS overaccumulation and oxidative stress in *W. anomalus*, concurrently enhancing the activities of antioxidant enzymes (SOD, CAT) and stimulating glutathione synthesis. This ethanol-triggered oxidative stress further compromised mitochondrial structure and function, ultimately reducing cell viability and promoting autophagy. Critically, antioxidant supplementation alleviated oxidative stress by restoring mitochondrial integrity and function, while attenuating ethanol-induced autophagy. These findings elucidate the mechanisms underlying ethanol toxicity in *W. anomalus* and indicate that exogenous antioxidants may enhance ethanol tolerance in this yeast.

## Supplementary Information

Below is the link to the electronic supplementary material.

Supplementary Material 1

## Data Availability

No datasets were generated or analysed during the current study.
