# Supplementary material for: Ethanol induced oxidative stress, mitochondrial dysfunction, and autophagy in Wickerhamomyces anomalus
Source: Microb Cell Fact. 2025 Nov 12;24:231. doi: 10.1186/s12934-025-02864-0 (PMC12613665; doi:10.1186/s12934-025-02864-0)
Supplement: Supplementary file 1 — Supplementary Material 1 [file 12934_2025_2864_MOESM1_ESM.docx]

**Supplementary materials**


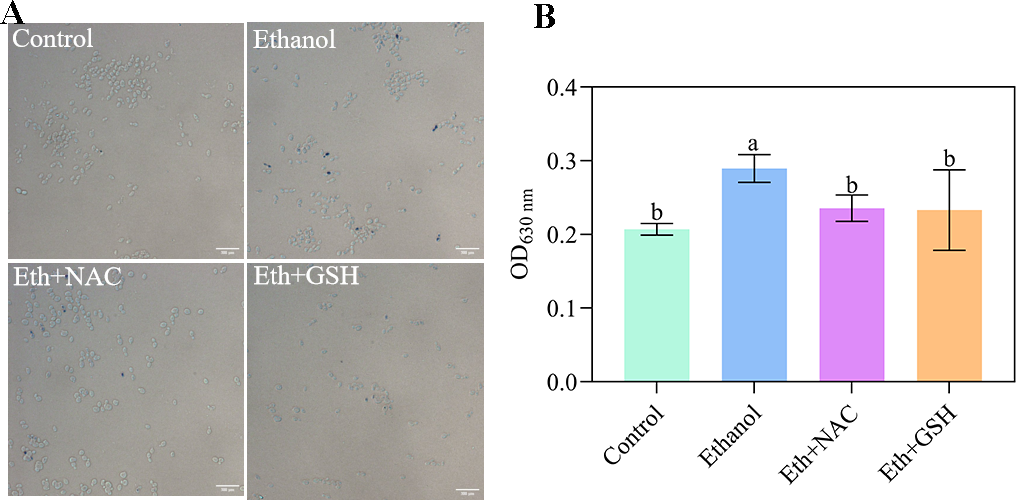


**Fig. S1** Results of superoxide anion (O_2_^·-^) detection by NBT staining. (A) Representative NBT staining images. (B) Quantification of NBT staining intensity measured at OD_630 nm_; Scale bar = 300 μm. Different lowercase letters above the error bars denote significant differences among treatments (P < 0.05).


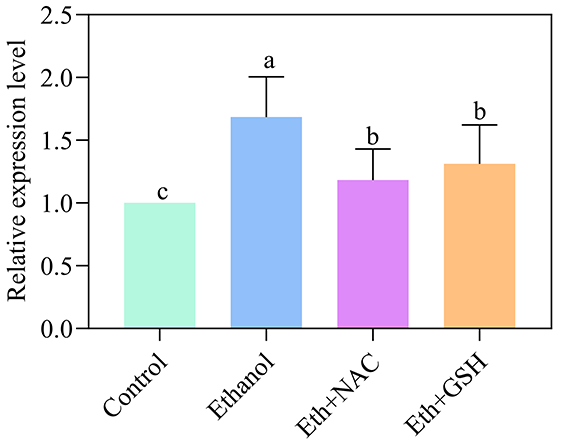


**Fig. S2** *ATG32* expression levels measured by qPCR using the primer sequences for were forward 5‘-CACGATTCCGCAACATTCCC-3’ and reverse 5’-ACTTGGATGACTTGCCCAGG’. Different lowercase letters above the error bars denote significant differences among treatments (p < 0.05).
